# Sialoadhesin (CD169/Siglec-1) is an extended molecule that escapes inhibitory *cis*-interactions and synergizes with other macrophage receptors to promote phagocytosis

**DOI:** 10.1007/s10719-022-10097-1

**Published:** 2023-02-04

**Authors:** Mariliis Klaas, Stuart Dubock, David J. P. Ferguson, Paul R. Crocker

**Affiliations:** 1 Division of Cell Signalling and Immunology, School of Life Sciences, University of Dundee, Dow Street, Dundee, United Kingdom; 2 Department of Cell Biology, Institute of Molecular and Cell Biology, University of Tartu, Riia 23, Tartu, Estonia; 3 Nuffield Division of Clinical Laboratory Sciences, Oxford University, John Radcliffe Hospital, Oxford, United Kingdom; 4 Department Biological & Medical Sciences, Oxford Brookes University, Oxford, United Kingdom

**Keywords:** Sialoadhesin, Siglec, Sialic acid, Adhesion, Macrophage, Phagocytosis

## Abstract

Sialoadhesin (CD169/Siglec-1, Sn) is a macrophage receptor that interacts with sialic acids on both host cells and pathogens. It is a type 1 membrane protein with an unusually large number of 17 extracellular immunoglobulin (Ig)-like domains, made up of an N-terminal V-set domain that binds sialic acid and 16 adjacent C2-set domains. The potential importance of 17 Ig domains in Sn for mediating cellular interactions has not been investigated experimentally. In the present study, Chinese Hamster Ovary (CHO) cells were stably transfected with full-length or truncated forms of Sn. Using human red blood cells (RBC) as a model system, CHO cells expressing truncated forms of Sn with 4 or less Ig domains were unable to bind RBC in comparison to the full-length protein. Immunoelectron microscopy of the CHO cells indicated that full-length Sn extends ~ 33 nm from the plasma membrane compared with ~ 14 nm for a truncated form with 6 N-terminal Ig domains. Co-expresssion of Sn-expressing CHO cells with heavily glycosylated membrane proteins of differing predicted lengths resulted in selective modulation of Sn-dependent binding to RBC and supported the hypothesis that Sn has evolved 17 Ig domains to escape inhibitory *cis*-interactions. The functional significance of the extended length of Sn was demonstrated in experiments with macrophages showing that Sn synergizes with phagocytic receptors FcR and TIM-4 to strongly promote uptake of IgG-opsonized and eryptotic RBC respectively.

**Supplementary Information:**

The online version contains supplementary material available at 10.1007/s10719-022-10097-1.

## Introduction

The immunoglobulin (Ig) superfamily plays a critical role in immune recognition at the cell surface via interactions with ligands expressed on host cells and pathogens. Most members of the Ig superfamily mediate protein-dependent heterotypic and homotypic interactions and typically have between 1 and 10 Ig-like domains [1]. The siglecs are a distinct subgroup of the Ig superfamily that mediate sialic acid-dependent carbohydrate recognition, through a homologous N-terminal V-set Ig domain [2]. Sialoadhesin (Sn) (also known as Siglec-1 and CD169) is the largest member of the siglec family and differs from other siglecs in having the unusually large number of 17 extracellular Ig domains, compared to between 2 and 7 Ig domains in other siglecs, [2–4].

The extracellular region of Sn is well conserved amongst mammalian species, suggesting its length is inherently important for functions mediated by the receptor. Furthermore, the carbohydrate binding properties and expression patterns of Sn are well conserved between rodents and humans, where it is expressed by macrophage subsets both under resting and inflammatory conditions [5, 6]. Unlike most siglecs, Sn can function as a cellular interaction molecule and mediate *trans* interactions with ligands on other cells and sialylated pathogens when expressed naturally at the cell surface [7–10]. In contrast, other siglecs of the immune system are usually masked by *cis*-interactions with sialic acids co-expressed on the same cell [4, 11].

It has been proposed that the extended length of Sn at the cell surface reduces *cis*-interactions of the V-set domain with ligands on the macrophage membrane and allows Sn to mediate sialic acid binding to ligands in *trans* [12]. This has been coined the ‘rainforest model’ by analogy to evolution of tall trees that extend above the underlying rainforest canopy, allowing access to sunlight [13]. However, no direct evidence to support this model has been reported. In the present study, using human RBC as a model system, we investigated the role of the length of Sn in cell-cell binding and its influence on preventing inhibitory *cis*-interactions. By using CHO cells transfected with a full-length construct or with truncated mutants and a combination of cell binding assays, immunoelectron microscopy and *cis*-competition assays with other glycosylated membrane proteins, our results provide experimental support for the rainforest model. To explore its functional significance, we used macrophages that either lack Sn or express a mutant form of Sn that is unable to bind sialic acid and demonstrate that Sn is required for efficient phagocytosis of either IgG-coated RBC or eryptotic RBC expressing phosphatidylserine. This indicates that Sn can interact synergistically with ‘professional’ phagocytic receptors, leading to more rapid clearance of opsonized or damaged cells.

## Materials and methods

All chemicals and reagents were purchased from Sigma-Aldrich unless otherwise specified. Restriction and modifying enzymes were from Pharmacia, Boehringer or Gibco/BRL.

### Generation of Chinese Hamster Ovary (CHO) cells expressing truncated forms of Sn

**Fig. 1:**
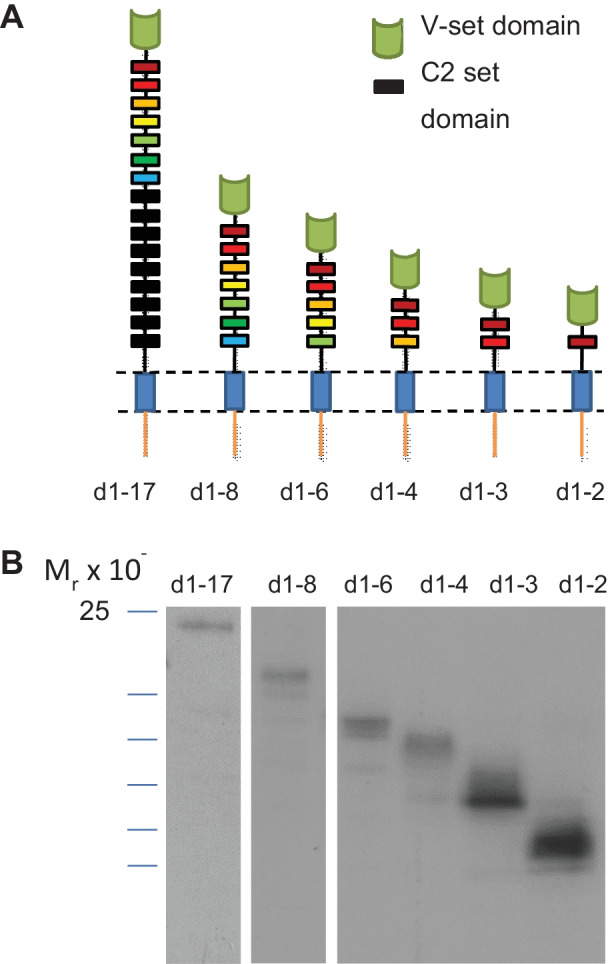
Expression of truncated forms of Sn in CHO cells. **A** Schematic diagram showing the domain organization of the full-length (d1-17) and the truncated forms of Sn. **B** Western blotting of CHO cell clones expressing the indicated number of Ig domains

A full-length cDNA of Sn in the pcDNA1/Amp plasmid was described previously [12] and used as a template for PCR reactions. A NsiI restriction site was introduced at a position corresponding to the C-terminal end of domain 17 of Sn by PCR using as forward primer: (5’-3’) ACATGCATCAACTGCAGCTGTTCCAGAGG with the SP6 reverse primer: ATTTAGGTGACACTATAG. The PCR product was cut with NsiI and XbaI and cloned into pcDNA1/Amp cut with NsiI and XbaI. Truncated deletion constructs Sn d1-8, Sn d1-6, Sn d1-4, Sn d1-3 and Sn d1-2 (Fig. 1) were generated by PCR using the T7 forward primer: (5’-3’) TAATACGACTCACTATAGGG with the following reverse primers: ACATGCATCACACTTAGGACCACAGGAGC (Sn d1-8), ACATGCATCACAGTGAGGACAGTAGGCAG (Sn d1-6), ACATGCATGACCACCACACTCAATGGACT (Sn d1-4), ACATGCATAAAAACATGGAGGCTGAGCGG (Sn d1-3) and ACATGCATCAGGTAAACCTCTTTCCGACT (Sn d1-2). PCR products were digested with HindIII and NsiI and cloned into HindIII and NsiI-digested pcDNA1 Amp containing the 3’ end of Sn cDNA encoding the transmembrane and cytoplasmic tail of Sn as described above. Expected products were confirmed by DNA sequencing.

CHO cells cultured in Hams F10 + 10% fetal calf serum (FCS) were stably transfected with cDNAs encoding the full-length d1-17 form of Sn or truncated constructs described above, following established protocols [14]. Clones expressing each construct were selected by limiting dilution and analysed by flow cytometry using 3D6 mAb (prepared in-house) directed to the N-terminal V-set domain of Sn. Clones in which > 95% of cells expressed Sn were selected for use in binding assays and co-transfection experiments. Western blotting was carried out using previously established methods [5] using SDS-PAGE and 5–15% gradient gels.

### Preparation of anti-Sn d1-specific antibody

A polyclonal rabbit antiserum was raised to native Sn purified from mouse spleens [15]. IgG specific for domain 1 of Sn (Sn d1) was isolated by affinity chromatography in 2 steps. First, total IgG was purified using a 1 ml Protein A Sepharose column (Pharmacia) following the manufacturer’s instructions and IgG specific for Sn d1 was purified using a 1 ml column of recombinant purified Sn d1 [16] coupled at 2.0 mg/ml to Sepharose CL4B (Pharmacia) as described [5].

### RBC binding assays and quantification of Sn expression on CHO cell truncated mutants

CHO cells expressing truncated constructs were seeded onto 24-well plates (Nunc) and grown to 80% confluence. Binding assays with human RBC were performed as described previously [17]. The total number of RBC bound per 100 CHO cells was determined by counting randomly selected fields in quadruplicate wells. To measure expression of Sn for the different constructs, anti-Sn d1 IgG described above was labelled with ^125^I (Amersham) as reported previously [5], diluted to 1 µg/ml in ice cold Hanks Buffered Hanks Solution + 0.1% bovine serum albumin and added to pre-chilled 24-well plates, using quadruplicate wells for each construct. After incubation for 1 h on ice, plates were washed 5 times with buffer and cell-associated radioactivity measured after solubilization in 0.1 M NaOH. Non-specific binding was determined using non-transfected wild-type CHO cells and was subtracted from test samples.

### Transmission immunoelectron microscopy

Anti-Sn d1 IgG was conjugated to 5 nm gold particles (British BioCell International) following the manufacturer’s instructions. For cell labelling, a pre-embedding immuno-staining technique was employed. Samples of wild-type CHO cells or CHO cells expressing the 17-, 6- or 2-domain form of Sn were fixed in 2% paraformaldehyde in 0.1 M phosphate buffer for 20 min at 4 °C and washed with Tris buffered saline. The samples were then incubated overnight in a suspension of anti-Sn d1 IgG gold conjugate, washed in buffer, fixed in 4% glutaraldehyde in 0.1 M phosphate buffer and processed for routine transmission electron microscopy [18]. Samples were post-fixed in 1% osmium tetroxide, dehydrated in an ethanol series, treated with propylene oxide and embedded in Spurr’s epoxy resin. Thin sections were stained with uranyl acetate and lead citrate prior to examination in a JEOL 1200EX electron microscope. Representative micrographs were taken at a magnification of x50,000. The distance of the gold particles from the plasma membrane was measured from photomicrographs using ImageJ software.

### Co-expression of CD43, MUC-1 and CR1 in CHO cells

Mouse CD43 cDNA in pCDM8 was a kind gift from Prof. P.A. van der Merwe, University of Oxford, Oxford, UK; human CR-1 cDNA in pCDM8 was a kind gift from Prof. D. Fearon, University of Cambridge, UK and human MUC-1 cDNA in pcDNA1/Amp was a kind gift from Prof. J. Taylor-Papadimitriou, Kings College London, UK. CHO cells expressing Sn d1-17 or Sn d1-6 were trypsinized, resuspended at 1 × 10^7^ cells/ml in phosphate buffered saline (PBS) and 1 ml aliquots mixed with 30 µg of CD43, MUC-1 or CR1 cDNA in electrocuvettes. Electroporation capacitance was set to 960 µF and the voltage to 220 volts. Electroporated cells were cultured on 15 cm cell culture dishes for 24 h and transferred to 8-well Lab-Tek chamber slides (Nunc) at 2 × 10^4^ cells per well and cultured in Hams F10 medium with 5% FCS and 2 mM sodium butyrate. RBC binding assays were performed at 72 h post-transfection and cells fixed for 10 min in 0.125% glutaraldehyde. After washing in PBS, non-specific binding sites were quenched using PBS + 10% FCS. Cell surface expression of CD43, MUC-1 and CR1 was determined by immunoperoxidase labelling using a Vectastain ABC kit with diaminobenzidine as substrate [17], using rat anti-mouse CD43 monoclonal antibody (mAb), clone S7 (Pharmingen), mouse anti-human MUC-1 mAb, clone SM3 (ICRF Hybridoma Facility) and mouse anti-human CR1 mAb, clone E11 (Serotec) The percentage of CHO cells binding more than 1 RBC was determined by microscopy using triplicate wells for each condition. On each well, RBC binding was evaluated for 100 cells that lacked expression of the co-transfected molecule (unstained by immunoperoxidase) and 100 cells that expressed it (stained by immunoperoxidase labelling).

### Mouse strains

Sn-deficient (Sn^−/−^) mice lacking Sn expression and Sn ‘knockin’ Sn^W2QR97A^ mice, expressing a mutant form of Sn unable to bind sialic acids, have been described previously [8, 19]. All mice used in experiments were inter-cross offspring of heterozygotes backcrossed for at least 8 generations onto a C57BL/6 background; age- and sex-matched mice at 8 to 15 weeks of age were used in experiments. Animals were housed in specific pathogen-free conditions.

### Isolation and culture of bone marrow-derived macrophages (BMDM)

Mice were euthanized by CO_2_ inhalation and femora and tibia were collected in DMEM (Gibco, Invitrogen). The marrow plugs were extruded with DMEM and disrupted by pipetting vigorously and passed through a 70 μm cell strainer. The bone marrow cells from each mouse were re-suspended in 15 ml complete growth medium: DMEM supplemented with 10% FCS (PAA Laboratories), 2 mM L-glutamine, 0.1 mg/ml streptomycin and 100 U/ml penicillin (Gibco, Invitrogen), with addition of 25 ng/ml murine M-CSF (PeproTech). The cells were cultured on 9 cm bacterial plastic Petri dishes (BD Biosciences) for 7 d and then lifted using 4 mg/ml lidocaine-HCl and 5 mM EDTA in PBS. After 7 d the BMDM were cultured for 3 d on either 8-well chamber slides (Nunc) at 6 × 10^4^ cells per well in 300 µl of complete growth medium supplemented with 250 U/ml IFN-α (PBL InterferonSource) or on 9 cm bacterial Petri dishes in 15 ml complete growth medium supplemented with 250 U/ml IFN-α.

### Immunostaining of cells and flow cytometry

Binding to FcRs (CD16/32) was blocked by incubating the cells on ice for 30 min with 2.4G2 hybridoma supernatant, except in experiments where the FcRs were stained when the blocking was performed by incubating the cells in 5% BSA in FACS wash solution (0.5% BSA, 2 mM NaN_3_, 2 mM EDTA in PBS). After blocking, the antibodies were added for 1 h: anti-Sn-biotin (3D6 and SER-4 mAbs produced in-house); anti-TIM-4-PE, anti-CD16/32-PE, anti-CD64-PE (all eBioscience); anti-FcγRIIB Ly17.2 (kind gift from Prof. J. Ravetch, The Rockefeller University, NY, USA) that was directly conjugated to Alexa 488 fluorescent dye using an antibody conjugation kit (Invitrogen). Corresponding isotypes were used at the same concentration in each staining. After incubating the cells with antibodies, the cells were washed in FACS buffer and, if necessary, incubated with streptavidin-APC (BD Bioscience, San Jose, CA, USA) for a further 30 min on ice and washed again. Cells were fixed with 1% formaldehyde in PBS and data were acquired with FACSCalibur (BD Bioscience). Analysis was performed using FlowJo 7.6.4 (Tree Star).

### RBC binding and uptake assays with BMDM

RBC (0.05% hematocrit in PBS) were opsonized using human anti-mouse glycophorin A (mouse IgG1, AbD Serotec) at a concentration of 1 µg/ml for 30 min at 37 ^◦^C and then treated with sialidase from *Vibrio cholerae* for 30 min at 37^◦^C in DMEM without FCS. 10% FCS was then added to cells to prevent further sialidase activity. After extensive washing in PBS, RBC were added to cells in complete growth medium at a density of 0.05% hematocrit, 300 µl per well. After allowing the RBC to settle for 20 min on ice, a phagocytosis assay was performed for 1 h at 37 ^◦^C. Bound, but not phagocytosed, RBC were lysed by hypotonic shock in H_2_O for 45 s. BMDM were fixed and permeabilized for 10 min on ice with methanol and stained with Diff-Quik staining kit (Dade Behring). In samples where the RBC were not lysed, the non-bound RBC were removed by washing before fixing the cells for 10 min in 0.125% glutaraldehyde in PBS. Finally, the slides were mounted into aqueous mounting media (National Diagnostics). Phagocytosis was quantified by counting the number of ingested RBC per 100 macrophages by light microscopy. Representative images were captured using a LSM 700 laser-scanning confocal microscope (Carl Zeiss) equipped with EC Plan-Neofluar 40 x / 1.30 Oil DIC M27 objective. Images were acquired with ZEN 2009 software (Carl Zeiss) and processed with ZEN 2009 Light Edition software (Carl Zeiss). Statistical analysis was performed using a paired two-tailed Student’s t test.

### *Escherichia coli* phagocytosis assays

After 7 d maturation with M-CSF, BMDM were seeded onto 12-well tissue culture plates at 5 × 10^5^ cells per well in 1 ml complete growth media and stimulated with 250 U/ml IFN-α for 72 h to induce Sn expression. The Alexa 488-labeled *E.coli* (Invitrogen, Molecular Probes) were opsonized for 1 h at 37 ^◦^C using *E. coli* opsonizing reagent (Invitrogen) according to the manufacturer’s recommendations. After opsonization, the concentrations of the opsonized bacteria and unopsonized controls were determined using a hemocytometer. The bacteria were added to BMDM at a ratio 1:10 (BMDM:bacteria) in 1 ml complete growth media. After letting the bacteria settle for 20 min on ice, the cells were placed in a 37 °C incubator for 1 h. To remove unbound bacteria, the cells were washed with PBS five times and lifted with PBS containing 4 mg/ml lidocaine and 5 mM EDTA. Finally, the cells were fixed with 1% formaldehyde and analyzed by flow cytometry using FACSCalibur. Unbound bacteria were excluded by gating on BMDM using FSC and SSC parameters. Statistical analysis was performed using a paired two-tailed Student’s t test.

### Isolation and culture of resident peritoneal macrophages (RPM)

Mouse RPM were obtained by lavaging the peritoneal cavity with 10 ml PBS containing 0.5% BSA and 2 mM EDTA. Cells were washed three times in PBS before resuspending in DMEM supplemented with 10% FCS, 2 mM L-glutamine, 0.1 mg/ml streptomycin and 100 U/ml penicillin. Cells were seeded on 8-well Lab-Tek chamber slides at 6 × 10^4^ cells per well in 300 µl of media. Non-adherent cells were rinsed off after letting the macrophages adhere for 2 h and used in RBC binding/phagocytosis assays. For flow cytometry-based assays, the cells were seeded on 4 cm bacterial Petri dishes (BD Falcon) and after 2 h the RPMs were lifted using 4 mg/ml lidocaine-HCl (Sigma-Aldrich) and 5 mM EDTA in PBS (Ca^2+^-, Mg^2+^-; Gibco, Invitrogen) and immunochemically labeled for analysis by flow cytometry.

### RPM binding and phagocytosis assays with eryptotic RBC

Eryptosis was induced in freshly collected human RBC as previously described [20]. Briefly, RBC were treated with 4 µM ionomycin for 1 h which induces a transmembrane calcium flux. Phosphatidylserine (PS) expression on the cell surface was measured using PE-conjugated annexin V apoptosis detection kit (BD Biosciences) according to the manufacturer’s instructions and cells were analyzed by flow cytometry. Treatment with *V. cholerae* sialidase and binding and phagocytosis assays with the eryptotic RBC were performed as described above for antibody-opsonized RBC. Statistical analysis was performed using a paired two-tailed Student’s t test.

## Results and discussion

### Effect of Sn truncation on cell binding activity

To investigate the significance of the 17 Ig-like domains of Sn in its function as a cell adhesion molecule, we generated stably transfected CHO cells expressing either full length (d1-17) or truncated forms of Sn (d1-8, d1-6, d1-4, d1-3 and d1-2) in which varying numbers of Ig domains were deleted (Fig. 1A). Western blotting confirmed the expected molecular weights for each truncated mutant (Fig. 1B) and flow cytometry using mAb 3D6, directed to the N-terminal domain, showed uniform high expression of Sn in each case (data not shown).

**Fig. 2:**
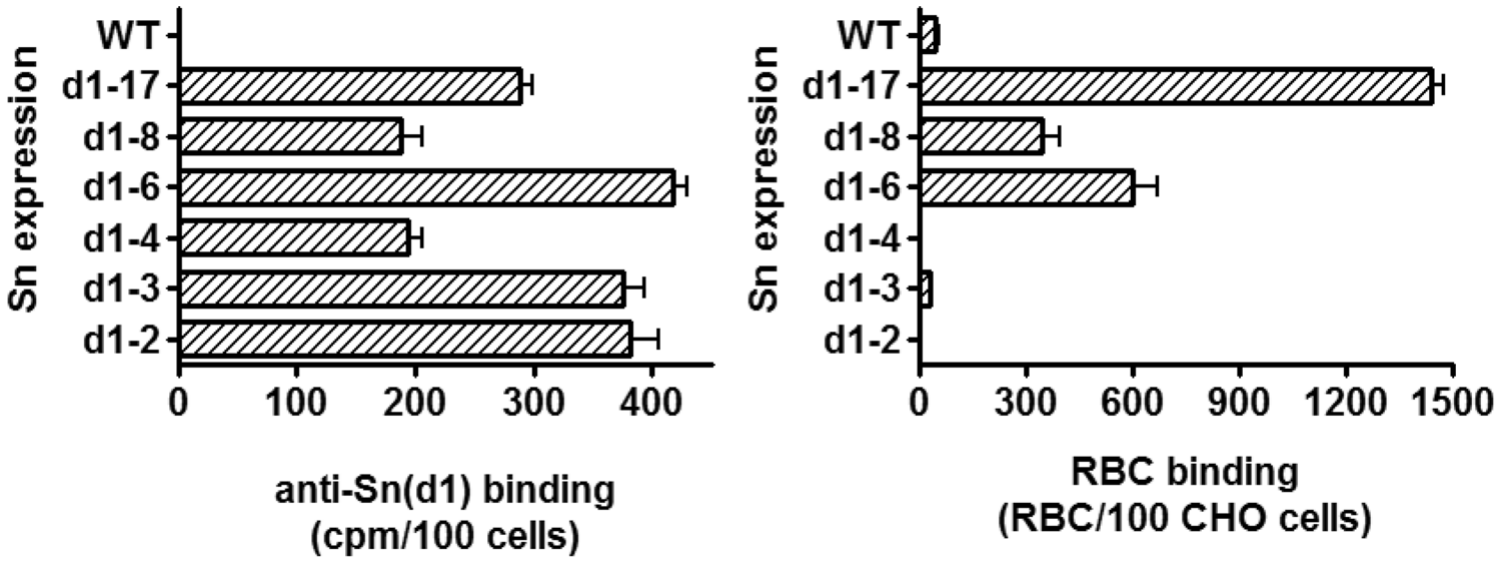
The effect of truncating Sn on RBC binding. The expression levels of the Sn truncated mutants on transfected CHO cells were quantified in binding assays using ^125^I radiolabeled anti-Sn d1-specific antibody (left panel). RBC binding assays were carried out with parallel cultures of CHO cells expressing truncated mutants (right panel). Error bars show the means ± SD (n = 4) from one experiment and are representative of 3 independent experiments carried out

We used human RBC as a model system to measure Sn-dependent cell adhesion and measured their binding to CHO cells expressing full-length and truncated mutants of Sn (Fig. 2). Highest RBC binding was observed with the full-length d1-17 form of Sn, but clear binding was also observed with CHO cells expressing 8 or 6 Ig domains. However, RBC binding to the 6-domain form was lower than that seen with CHO cells expressing the full-length form, despite expressing higher levels of Sn (Fig. 2). No RBC binding above background was observed using cells expressing the 4-, 3- or 2-domain forms of Sn, despite high levels of surface expression.

**Fig. 3:**
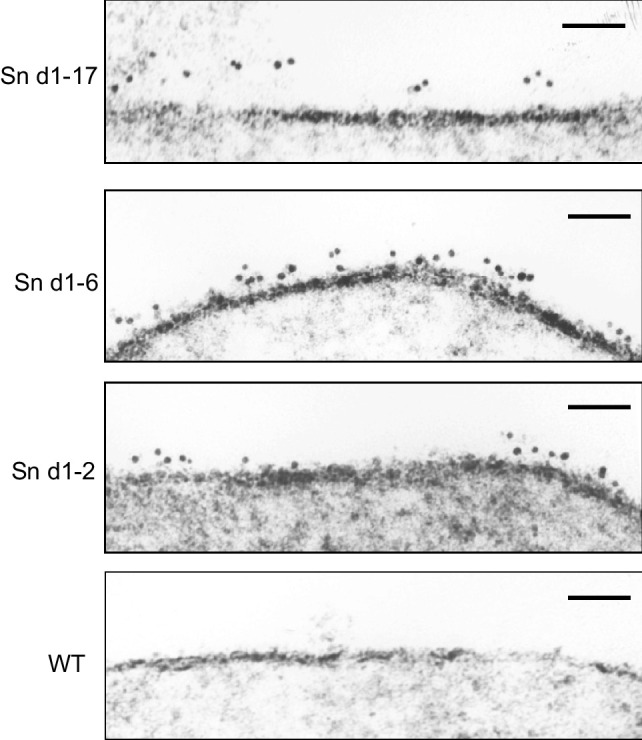
Visualisation of Sn extension from the plasma membrane by immunoelectron transmission microscopy. Immunoelectron micrographs showing cross sections through the plasma membrane of wild-type (WT) CHO cells and CHO cells transfected with the indicated forms of Sn deletion mutants. Post fixation, cells were labelled with anti-Sn d1-specific antibody, directly conjugated to gold particles. Representative images from 2 independent experiments are shown. Scale bars correspond to 50 nm

**Fig. 4:**
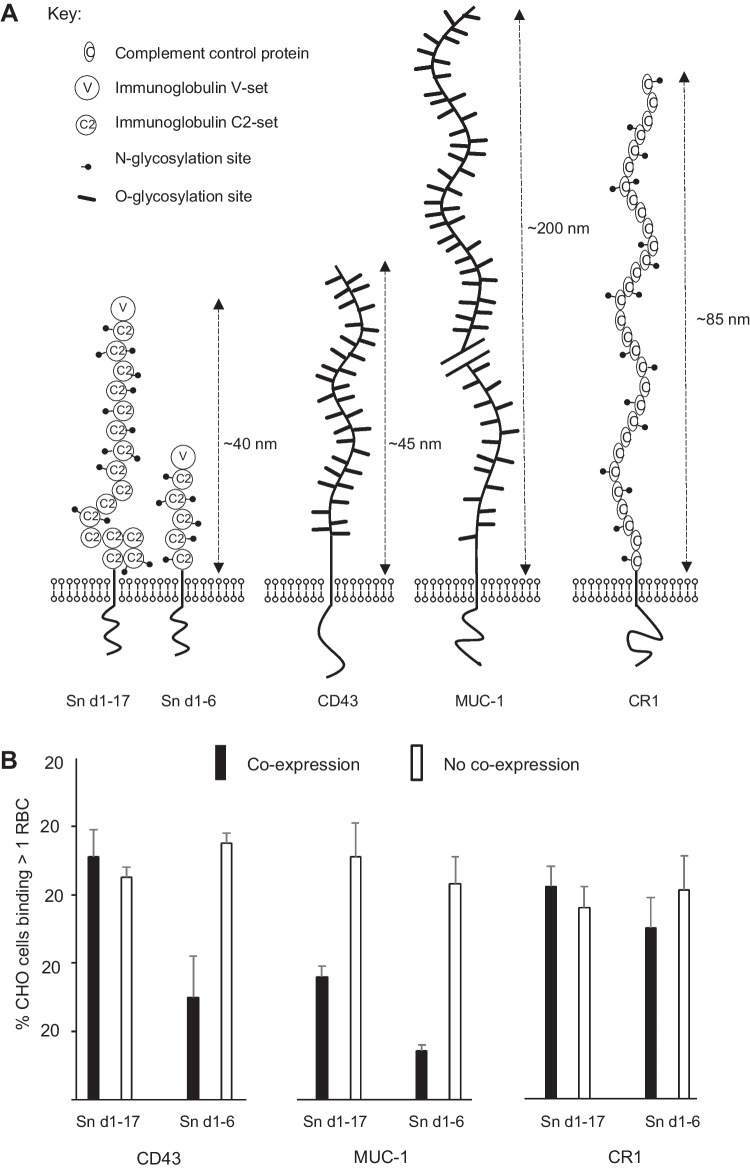
The effect of co-expression of glycoproteins of different length on binding of Sn to RBC. **A** Schematic diagram illustrating approximate molecular dimensions of Sn d1-17 and Sn d1-6 and other membrane proteins used in competition assays for modulation of Sn-dependent binding. Model for Sn is based on rotary shadowing and electron microscopy data showing that Sn is an extended tadpole-like structure with a globular region of ~ 9 nm and an extended tail of ~ 35 nm [15]. Models for CD43, MUC-1 and CR1 are based on published data [21–23]. **B** RBC binding assays were performed using CHO cells stably expressing the d1-17 full-length or d1-6 truncated form of Sn following transient transfection with CD43, MUC-1 or CR-1 cDNAs. RBC binding to CHO cells was quantified by counting the percentage of CHO cells binding > 1 RBC both on cells expressing and not expressing CD43, MUC-1 or CR1 cDNA in each culture. Expression was visualized by immunoperoxidase labelling with specific antibodies. Bars show the means ± SD of 1 experiment (n = 3) representative of 3 independent experiments carried out

To directly visualize extension of Sn from the cell surface, we produced immunogold conjugates of a polyclonal antibody directed to the N-terminal V-set domain of Sn and performed immunoelectron microscopy on wild-type CHO cells and CHO cells expressing the 17-, 6-, and 2-Ig domain forms of Sn (Fig. 3). Measurements of the distance of the gold particles from the plasma membrane showed that Sn d1-17 extended 32.9 +/- 6 nm (mean +/- SD), more than twice the distance of Sn d1-6 construct (14.1 +/- 4.2 nm) and around 3 times further than the Sn d1-2 construct 2 (9.6 +/- 5 nm). This may be an underestimate of the true distance on living cells due to shrinkage effects of the processing for electron microscopy. Indeed, purified native Sn was shown previously by rotary shadowing electron microscopy to be a tadpole-shaped structure, with a globular head of ~ 9 nm at the juxtamembrane region and an extended tail of ~ 35 nm [15]. Taken together with the current findings, this supports the notion that full-length Sn projects its N-terminal binding site up to ~ 40 nm above the plasma membrane (Fig. 4A).

### Co-expression with mucin-like sialoglycoproteins modulates Sn-dependent adhesion

Similar to other siglecs, the reduced or absent RBC binding seen with truncated forms of Sn is likely to be due to inhibitory *cis* interactions with glycoproteins co-expressed on the same cells [4]. To test this hypothesis, CHO cells stably transfected with either the full-length (d1-17) or truncated (d1-6) Sn were transiently co-transfected with various plasmid cDNAs encoding glycoproteins of defined lengths (Fig. 4). This resulted in mixtures of cells, all of which expressed Sn but only a fraction (~ 20–50%) expressed the co-transfected cDNA. By scoring RBC binding activity on the co-expressing and non-co-expressing CHO cells within the same culture, the influence of co-expressed molecules on RBC binding activity could be evaluated (Fig. 4B).

CD43 is a mucin-like glycoprotein expressed by macrophages that is heavily O-glycosylated and can extend up to 45 nm [21]. Co-expression of CD43 with Sn d1-6 led to considerable inhibition of RBC binding but no effect was seen on binding to the Sn d1-17 construct (Fig. 4B). MUC-1 is an O-glycosylated membrane mucin normally found on epithelial cells which can extend > 200 nm from the cell surface [22]. Co-expression of MUC-1 with Sn-expressing CHO cells led to a clear reduction in RBC binding to both the full-length and Sn d1-6 constructs (Fig. 4B). Finally, it was found that co-expression of complement receptor type 1 (CR-1/CD35), a membrane protein expressed by macrophages of similar length to CD43 but with much less glycosylation [23], did not affect RBC binding to the full-length or the d1-6 truncated form of Sn (Fig. 4B). In conclusion, these competition experiments are consistent with an important role of the length of Sn in allowing cell-cell binding in the presence of heavily glycosylated membrane glycoproteins.

### Sn-dependent uptake of IgG-opsonized RBC by BMDM

**Fig. 5:**
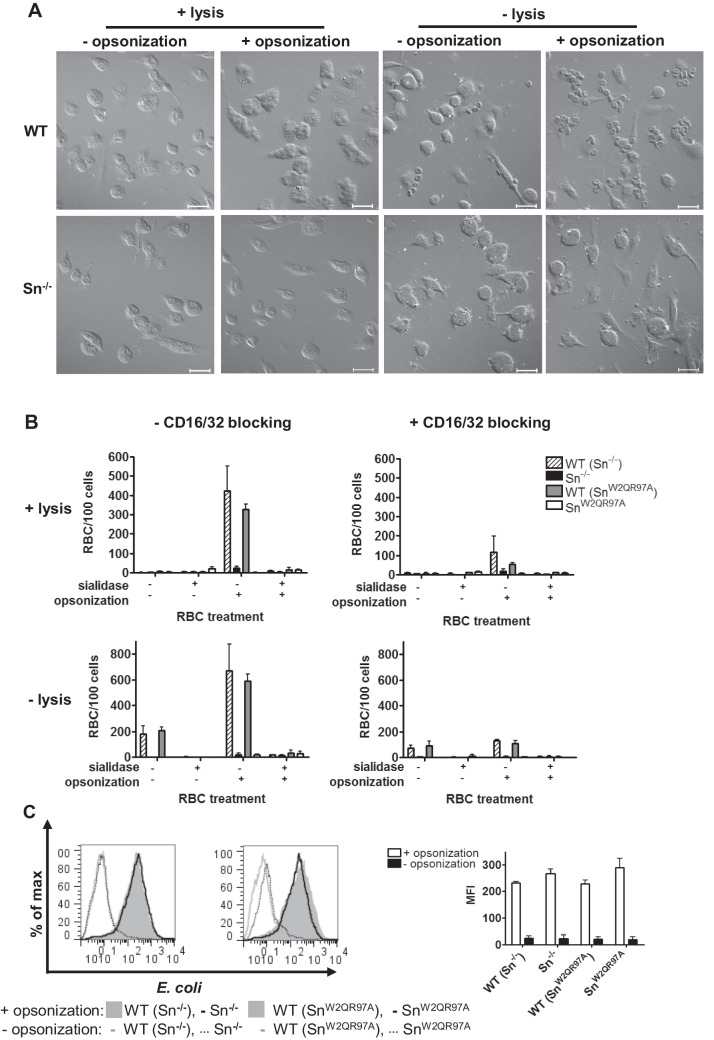
Sn-dependent uptake of IgG-opsonized RBC by BMDM. Representative images **A** and quantification **B** of uptake and binding of RBC, with or without opsonization and sialidase treatment, by WT, Sn^−/−^ and Sn^W2QR97A^ BMDM. Scale bars are 20 μm. Extracellular RBC were either fixed to BMDM surface (- lysis) or lysed with H_2_O (+ lysis). **B** RBC phagocytosis was blocked by mAb to CD16/32. Bars show the mean values of 3 independent experiments (3 replicates) ± SD. **C** Fluorescently-labeled *E. coli* were used as a positive control to demonstrate equal uptake of opsonized particles in WT, Sn^−/−^ and Sn^W2QR97A^ BMDM. One representative experiment (left panel) and data pooled from 2 independent experiments (right panel) are shown

To investigate the functional significance of the sialic acid-dependent adhesive properties of Sn in macrophage recognition, we explored the possibility it could synergize with phagocytic receptors of macrophages. Previous work established that Sn does not directly mediate phagocytosis in macrophages, and RBC bound to Sn-expressing macrophages remained at the cell surface for several hours in culture [17]. Many phagocytic receptors on macrophages, such as FcR, have small extracellular regions relative to Sn [24], raising the possibility that if potential targets co-express ligands for both Sn and a given phagocytic receptor, the extended length of Sn could promote cell-cell interactions and increase the efficiency of phagocytosis. To investigate a potential role of Sn in promoting FcR-mediated phagocytosis of IgG-opsonized RBC, assays were carried out using BMDM prepared from wild-type (WT), Sn^−/−^ and ‘knockin’ mice expressing a non-sialic-acid-binding mutant form of Sn (Sn^W2QR97A^) (Fig. 5). After lysis of extracellular RBC, it was found that WT BMDM phagocytosed high numbers of opsonized RBC while, in contrast, Sn-deficient BMDM showed a significantly lower ability to phagocytose the RBC (Fig. 5A). Furthermore, the uptake of opsonized RBCs by WT BMDM was almost abolished after sialidase treatment of the RBC, confirming that phagocytosis was sialic acid-dependent. The requirement of FcR in mediating phagocytosis was investigated using a blocking antibody, 2.4G2, which strongly reduced phagocytosis in WT BMDM (Fig. 5B). The failure of BMDM from Sn^−/−^ and Sn^W2QR97A^ mice to phagocytose IgG-opsonized RBC was not due to a defect in expression of FcR since they were able to phagocytose IgG-opsonized *E. coli* to the same extent as WT BMDM (Fig. 5C) and they expressed similar levels of FcR as determined by flow cytometry with 2.4G2 (Supplementary Fig. 1).

In contrast to opsonized RBC, phagocytosis of non-opsonized cells was not observed. However, in the absence of lysis, WT BMDM were seen to bind non-opsonized RBC, confirming that Sn mediates adhesion but does not function alone as a phagocytic receptor under the conditions used. In conclusion, these results clearly show that Sn can potently synergize with FcR for greatly increased efficiency of RBC uptake. This could be important in a wide range of inflammatory and infectious diseases where antibody-dependent clearance by macrophages is required for resolution. Sn is strongly upregulated by type I interferons, for example induced by bacterial or viral products or during inflammatory reactions [25] and may allow for efficient clearance of both ‘self’ as well as ‘non-self’.

### Sn-dependent uptake of eryptotic RBC by RPM

**Fig. 6:**
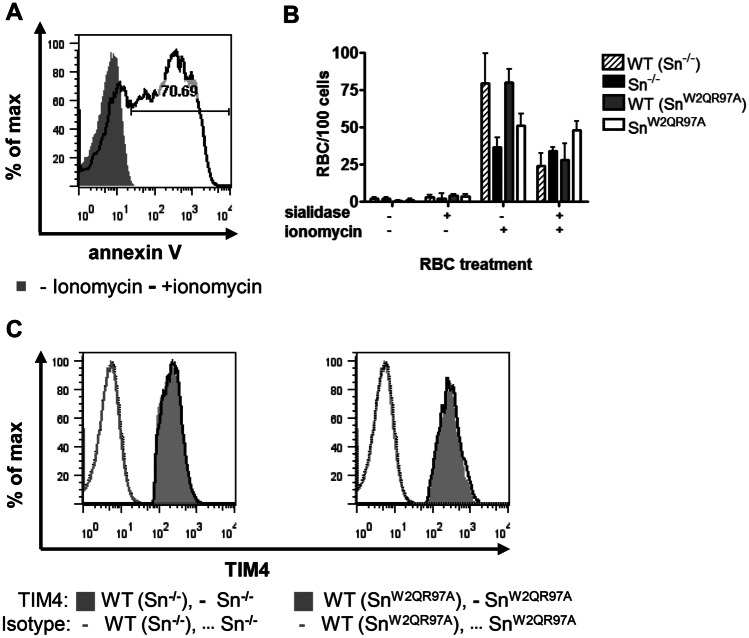
Sn-dependent uptake of eryptotic RBC by RPM. **A** Fresh untreated RBC or RBC made eryptotic by treatment with ionomycin were stained with annexin V to show expression of phosphatidyl serine at the cell surface. Data shown are representative of 3 independent experiments. **B** Uptake of RBC, with or without ionomycin and sialidase treatment, by WT, Sn^−/−^ and Sn^W2QR97A^ RPM. Bars represent the mean values of 3 experiments ± SD. **C** Cell surface expression of TIM-4 on WT, Sn^−/−^ and Sn^W2QR97A^ RPM was quantified by flow cytometry. One of 2 experiments is shown

Another important phagocytic response of macrophages is in the clearance of apoptotic cells expressing PS at the cell surface [24]. One of the major phagocytic receptors for PS is TIM-4, which is expressed by resident peritoneal macrophages (RPMs) [20, 26]. To investigate whether Sn could synergize with TIM-4 for phagocytosis, eryptosis or apoptosis-like cell death was induced in RBC by incubation with ionomycin, resulting in ~ 70% of RBC expressing PS at the cell surface (Fig. 6A). Using RPM from wild type and mutant mice, phagocytosis assays were carried out with untreated and eryptotic RBC, with or without sialidase pretreatment (Fig. 6B). After lysis of non-phagocytosed RBC, eryptotic RBC were significantly more phagocytosed by WT than Sn^−/−^ or Sn^W2QR97A^ RPM (Fig. 6B). In addition, the Sn-dependent phagocytosis could be reversed with sialidase treatment of RBC, showing a sialic acid-dependent effect. The reduced uptake of eryptotic RBC by mutant RPM was not due to altered TIM-4 expression, which was shown to be present at similar levels on cells from WT, Sn^−/−^ and Sn^W2QR97A^ mice (Fig. 6C). These results indicate that receptors for apoptotic cells, such as TIM-4, might potentially synergize with Sn to mediate phagocytosis of apoptotic cells and cellular debris.

Interestingly, TIM-4 has a similar expression profile to Sn, being highly expressed in strongly Sn-positive marginal metallophilic macrophages in spleen [27]. These macrophages have an important role in suppressing immune responses to apoptotic cell-associated antigens, but a direct role for Sn *in vivo* has not yet been investigated [28, 29]. Further studies are needed to determine whether Sn binding to sialylated ligands on apoptotic cells can promote uptake in synergy with phagocytic receptors such as TIM-4 and lead to more efficient clearance of ‘self’ antigens.

**Fig. 7:**
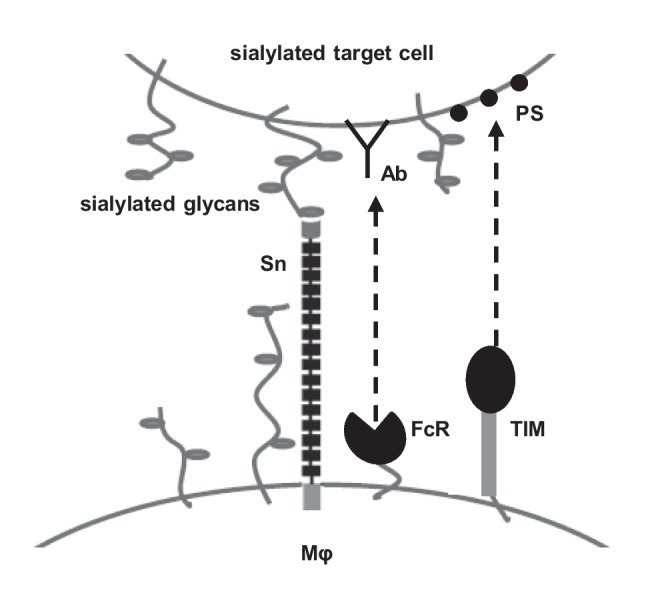
Model of Sn-mediated cell-cell binding and synergy with phagocytic receptors. The extended structure of Sn allows escape from inhibitory cis-interactions with sialoglycans on the macrophage to permit trans-binding to sialic acids present on target cells. Sn-mediated cell-cell binding then promotes interactions between macrophage phagocytic receptors and their ligands on target cells, e.g. FcRs that bind antibodies or TIMs that recognize PS

In conclusion, the data presented in this paper support our hypothesis that the extended length of Sn, comprising 17 Ig-like domains, is important for escaping the inhibitory influence of *cis*-interacting sialic acids presented by abundant mucins such as CD43. Thus, the extended nature of Sn could be important in promoting the initial contacts of macrophages with sialylated cells and pathogens, thereby increasing the efficiency of other less accessible phagocytic receptors on the macrophage surface (Fig. 7). Further studies are required to determine the *in vivo* significance of these observations in autoimmune responses, infectious disease and clearance of apoptotic cells.

## Electronic supplementary material

Below is the link to the electronic supplementary material.

Supplementary Material 1

## Data Availability

The datasets generated during and/or analysed during the current study are available from the corresponding author on reasonable request.
