# Supplementary material for: Sialoadhesin (CD169/Siglec-1) is an extended molecule that escapes inhibitory cis-interactions and synergizes with other macrophage receptors to promote phagocytosis
Source: Glycoconj J. 2023 Feb 4;40(2):213–23. doi: 10.1007/s10719-022-10097-1 (PMC10027830; doi:10.1007/s10719-022-10097-1)
Supplement: Supplementary file 1 — Supplementary Material 1 [file 10719_2022_10097_MOESM1_ESM.pdf]

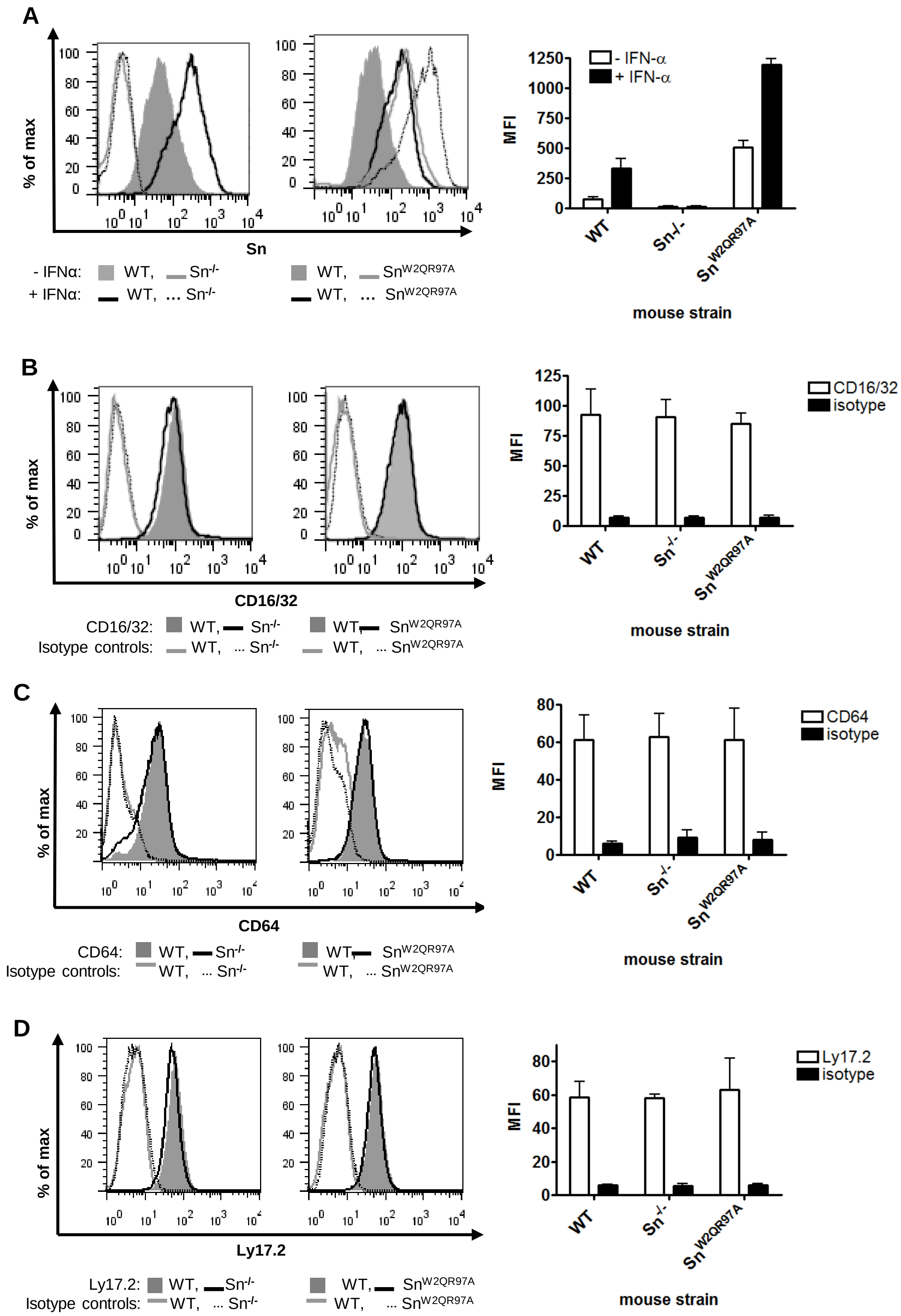

Supplemental Fig. 1. Expression of Sn and FcγRs on BMDM. BMDM from WT, $Sn^{-/-}$ and $Sn^{W2QR97A}$ mice were cultured with IFN-α to stimulate Sn expression (A) and stained with anti-CD16/32 (B), anti-CD64 (C), Ly17.2 (D), or an isotype matched mAb. Representative experiments (left panels) and the summary from 3 independent experiments (right panels) are shown.
